# A comprehensive murine clinical model for development of countermeasures and studying Mayaro virus infection

**DOI:** 10.1371/journal.pntd.0013333

**Published:** 2025-07-31

**Authors:** Rafael Borges Rosa, Emilene Ferreira de Castro, Débora de Oliveira Santos, Willyenne Marília Dantas, Camila Ayumi Tanaka, Renata Pessôa Germano Mendes, Ronaldo Celerino da Silva, Cláudio Antônio de Moura Pereira, João Paulo Silva Servato, Anaíra Ribeiro Guedes Fonseca Costa, Roberta Vieira de Morais Bronzoni, Lindomar José Pena

**Affiliations:** 1 Department of Virology and Experimental Therapy, Aggeu Magalhães Institute, Oswaldo Cruz Foundation, Recife, Brazil; 2 Postgraduate Program in Microbiology, Parasitology and Pathology, Federal University of Parana, Curitiba, Brazil; 3 Dental Hospital, Oral Pathology Laboratory, University of Uberlandia, Uberlandia, Brazil; 4 Clinical Hospital, Faculty of Medicine, University of São Paulo, São Paulo, Brazil; 5 Department of Dentistry, Uberaba’s University, Uberlandia, Brazil; 6 Federal University of Mato Grosso, Institute of Health Sciences, Sinop, Montana, Brazil; UCLM: Universidad de Castilla-La Mancha, SPAIN

## Abstract

The Mayaro virus (MAYV) is an arthropod-borne virus that causes Mayaro fever, a neglected tropical disease that produces disabling arthralgia. Given the significant threat the dissemination of MAYV poses to global public health, the development of animal models for the Mayaro fever could help elucidate its pathogenic mechanisms and routes of transmission and support the production of prophylactic and therapeutical agents. Thus, this work aimed to characterize a susceptible murine model for MAYV infection. Type I IFN receptor knockout (A129 KO) and wild-type 129S1 mice (A129 WT), 21 days old and from both sexes, were inoculated with the MT/SINOP/210/2011 Brazilian MAYV strain in the footpad, with phosphate-buffered saline-inoculated animals as controls. Clinical signs of infection, survival, body temperature, weight loss, paw swelling, hematological changes, viral load in solid organs and serum, as well as histopathological changes in the tibiotarsal joints were evaluated. MAYV animal models have not been extensively studied using the hypernociception and loss of muscle strength analysis system, therefore we also performed the Von Frey and Kondziella tests. MAYV infection triggered a systemic disease in KO male mice, while local pain and loss of muscle strength were more evident in females. Survival was lower in the KO group than in the WT animals. Both the Von Frey and Kondziella tests showed superior sensitivity in detecting local clinical signs of infection compared to footpad thickness measurements. A marked lymphocytic inflammatory response was observed in the tibiotarsal joints of KO animals, who had increased footpad thickness compared to the WT group. Higher viral titers were detected in the joints and associated muscles of KO mice compared to the WT group at 3 d.p.i., as well as in the brain and gonads of WT and KO animals at 6 d.p.i. In conclusion, we demonstrated that A129 KO mice are efficient in replicating the main clinical signs of the disease caused by Mayaro virus. The Brazilian strain might be neuropathogenic and sexually transmitted, showing that the Mayaro fever might be a serious health care concern.

## Author summary

The Mayaro fever is an infectious disease transmitted by mosquitoes that causes disabling joint pain and swelling. Seventy years after the first cases were detected, the Mayaro virus (MAYV) is considered a global health issue due to the elevated risk for urban dissemination. Besides the efforts for controlling vector populations, vaccines, and antivirals must be developed to prevent and treat the Mayaro fever. This is only possible when studies are conducted to understand the disease, usually with the aid of laboratory animals. In this sense, research must be performed to identify appropriate animal models for human illness. In this work, we infected immunocompetent and immunodeficient laboratory mice with a clinical isolate of MAYV. We observed that the immunodeficient animals manifested more signs of the disease and would be appropriate for studying MAYV. Interestingly, systemic signs of infection were more evident in male mice, while females suffered more from joint pain and muscle weakness. We have also detected the virus in the brain and gonads, showing that MAYV can affect the nervous system and be sexually transmitted. Our study proposes a novel animal model that could be used to investigate MAYV biology, transmission, and possible preventive and therapeutical agents.

## Introduction

Mayaro virus (MAYV), a member of the *Togaviridae* family and the *Alphavirus* genus, is the etiological agent of the Mayaro fever a mild, self-limiting febrile syndrome similar to dengue and chikungunya. The disease has two phases: acute and convalescent [1,2]. The acute phase is characterized by a short and transient viremia. Following the incubation period, fever suddenly develops, ranging from 39° to 40.2°C [3], and is accompanied by symptoms such as frontal headache, arthralgia, myalgia, joint swelling, chills, retro-orbital pain, malaise, rash, vomiting, and diarrhea [1,4,5]. In some cases, additional symptoms may include nausea, cough, sore throat, abdominal pain, nasal congestion, itching, anorexia, swollen lymph nodes, and bleeding gums [1,3,5]. Approximately 20% of the cases present with swelling of the small joints, particularly in the wrists, fingers, ankles, and toes [3]. After the acute phase, the convalescence phase begins, which may be accompanied by persistent arthralgia and arthritis, lasting for several weeks or even months [2,3,6].

MAYV was first isolated in Trinidad and Tobago in 1954 and has since been reported in several countries across the tropical regions of South and Central America. Although MAYV has an enzootic cycle, it can infect humans. However, inadequate surveillance and the non-specific nature of the clinical manifestations often result in misdiagnosis with other viral fevers [7]. Since its discovery, sporadic cases, outbreaks, and small epidemics of Mayaro fever have been reported. Clinical cases of this febrile illness and virus isolation have been reported in Brazil, Peru, Suriname, French Guiana, Guyana, Venezuela, Colombia, Ecuador, Panama, and Bolivia. Serological studies also suggest the presence of the virus in Costa Rica, Guatemala, and Mexico [7,8].

MAYV can infect, replicate, and spread in both vertebrate and invertebrate hosts. In endemic regions of Latin America, the transmission cycle primarily occurs in wild and rural areas. In this type of cycle, the hematophagous mosquito *Haemagogus janthinomys* is considered the primary vector, while non-human primates serve as the primary hosts. However, to a lesser extent, other types of mosquito species, such as those of the genus *Mansonia* and *Culex*, may also act as occasional vectors. Some vertebrates, including rodents, reptiles, and birds, can serve as viral reservoirs Human infection is considered accidental and occurs when individuals are exposed to wild reservoir habitats [9–11].

Phylogenetic analysis of MAYV based on the E2/E1 gene sequence identifies three distinct genotypes. The genotype D (disseminated) is widespread in South America, while genotype N (new) is exclusive to Peru, and genotype L (limited) is primarily confined to northern Brazil, first isolated in Pará in 1955 [12]. The presence of genotype L has been reported in central-west Brazil together with a similar strain in Haiti, suggesting recombination between strains [13].

MAYV belongs to the Semliki virus complex, alongside chikungunya virus (CHIKV), a well-established urban pathogen. Studies have shown that *Aedes aegypti* can transmit MAYV [14], with infections reported near large cities infested by this vector [12]. The potential for the vector to adapt to urban environments, coupled with anthropogenic factors such as rapid urbanization, climate change, and increased population mobility, heightens the risk of the virus spreading to other continents and causing outbreaks in previously unaffected regions.

Appropriate animal models replicate Mayaro fever similarly to the human disease, providing insights into the virus’s biology and infection mechanisms. Despite the limitations of species specificity *in vivo* experiments, murine models have been widely used and have significantly contributed to biomedical research on urban arboviruses of public health concern [15]. While previous studies on MAYV using murine models have focused on elucidating viral mechanisms and testing vaccines and drugs, this work is dedicated to the robust characterization of the model and the detailed description of MAYV pathogenesis in mice. Specific tests were employed to replicate the main phenotypes observed in human patients with Mayaro fever. Additionally, this study evaluates pathogenesis in both sexes using, for the first time, a Brazilian isolate of MAYV (MT/SINOP/210/2011).

## Results

### MAYV infection causes hypothermia in A129 KO mice

**Fig 1:**
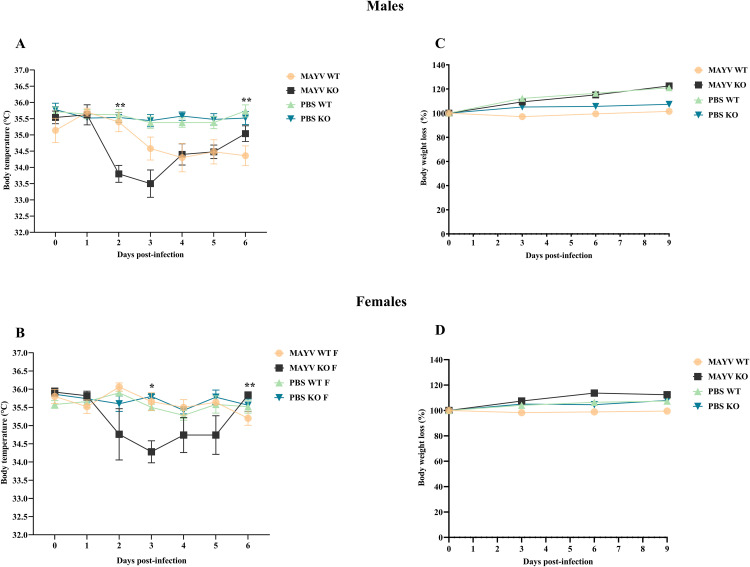
MAYV infection causes hypothermia in A129 KO mice but is not associated with weight loss in this murine model. **(A)** The body temperature of MAYV-infected knockout (MAYV KO) males significantly reduced when compared to both infected wild-type (MAYV WT) animals at 2 days post-infection (d.p.i.) and controls (PBS KO) at 2 and 5 d.p.i. **(B)** MAYV KO females had significantly lower temperatures when compared to WT mice only at 3 d.p.i. (C) and **(D)** No significant differences between infected and control animals regarding weight loss were found. Two-way ANOVA with Tukey’s multiple comparisons tests.

In contrast to the high-grade fever typical in the human infection, experimentally infected mice had transient hypothermia (Fig 1). This body temperature reduction was observed in immunocompetent wild-type (A129 WT) males but more prominently in immunodeficient (A129 KO) mice. In KO mice, hypothermia began on the first day post-infection (d.p.i.), dropping to 2°C at 3 d.p.i., then gradually increasing after 4 d.p.i. Wild-type male mice experienced a milder 1°C decrease in body temperature between 3 and 6 d.p.i. Significant differences were observed between infected WT and KO males at 2 d.p.i. and between KO males (MAYV and PBS) at 2 and 5 d.p.i. (Fig 1A).

In KO females, body temperature reduction began later (2 d.p.i.) than in males, reaching a peak drop of 1.5°C at 4 d.p.i. and returning to baseline by 6 d.p.i. Wild-type females exhibited temperature fluctuations of ± 1°C throughout the evaluation period. Significant differences between WT and KO females were observed only at 3 d.p.i. (Fig 1B).

### MAYV infection does not cause weight loss in this murine model

MAYV-infected Immunocompetent and immunodeficient mice maintained their body weight regardless of sex. Although the KO animals developed overt disease, this was not a determining factor for weight loss. No significant differences were observed between infected and control groups or between sexes throughout the 10-day evaluation (Fig 1C and 1D).

### MAYV infection is lethal for A129 KO mice

**Fig 2:**
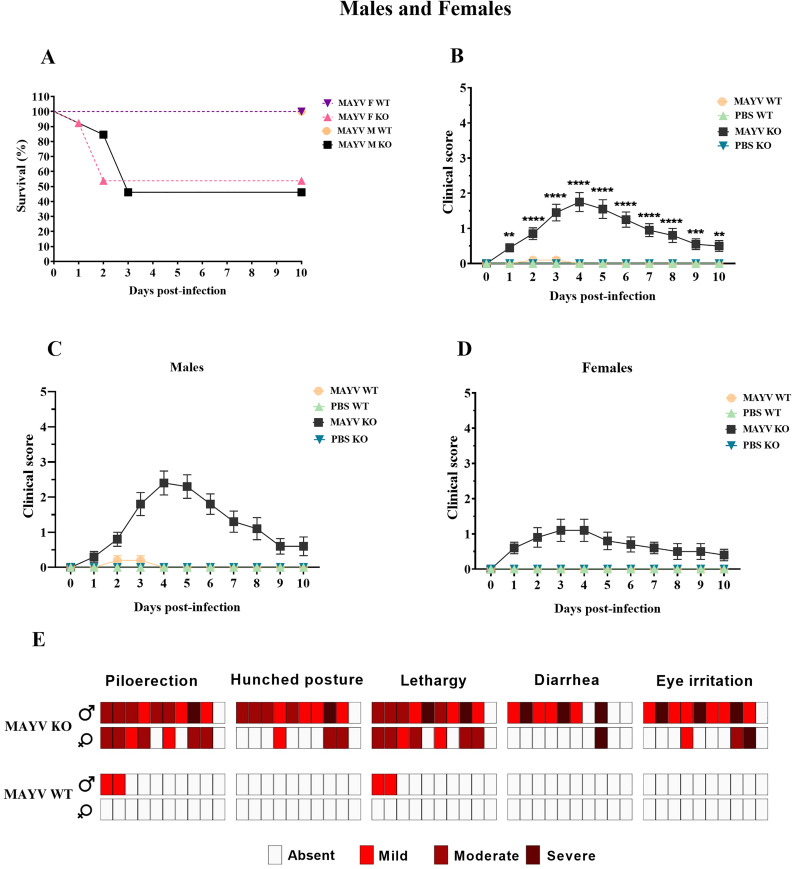
MAYV infection is lethal for A129 KO mice, who manifest more evident clinical signs of the disease. **(A)** Survival analysis of infected wild-type (MAYV M WT) and knockout (MAYV M KO) male mice and infected wild-type (MAYV F WT) and knockout females (MAYV F KO) using the Mantel-Cox log-rank test. Survival of KO animals significantly reduced when compared to the WT group (*p* < .0001). KO animals show higher scores in the clinical evaluation when compared to WT and control groups in the (B) sex-independent analysis. Clinical signs of infection are more prominent in KO males (C) when compared to KO females **(D)**. Two-way ANOVA with Tukey’s multiple comparisons tests. **(E)** The severity of the clinical signs observed in MAYV-infected mice. A high number of MAYV KO males expressed severe phenotypes compared to MAYV KO females and MAYV WT animals.

The MAYV infection progression was lethal for immunodeficient animals, with higher mortality in males KO (54%) than females KO (46%) Seven males died between 2 and 3 d.p.i., while six females died between 1 and 3 d.p.i. (Fig 2A). No deaths were observed in the infected immunocompetent animals or in the control groups (KO or WT).

### A129 KO mice express overt disease phenotypes

MAYV-infected immunodeficient mice exhibited a clear clinical progression of the disease, with evident manifestation of phenotypes (Fig 2B). Males displayed more pronounced clinical signs, ranging from moderate to severe intensity (Fig 2C). Animals started to manifest overt disease at 2 d.p.i., peaking between 5 and 6 d.p.i. In the acute phase of infection, there was an increase in lethargy, piloerection, eye irritation with secretion, diarrhea, and hunched posture, representing a three-point score in the clinical assessment (Fig 2C) (S1 Table).

On the other hand, female KO mice reached a clinical score of 2, exhibiting increased lethargy, piloerection, eye irritation, diarrhea, and hunched posture. Besides the lower intensity of the clinical signs, which varied from mild to moderate, a higher proportion of asymptomatic animals was observed in the female group compared to males (Fig 2D). Overt disease was observed at 1 d.p.i., albeit more evident between 3 and 4 d.p.i.

**Fig 3:**
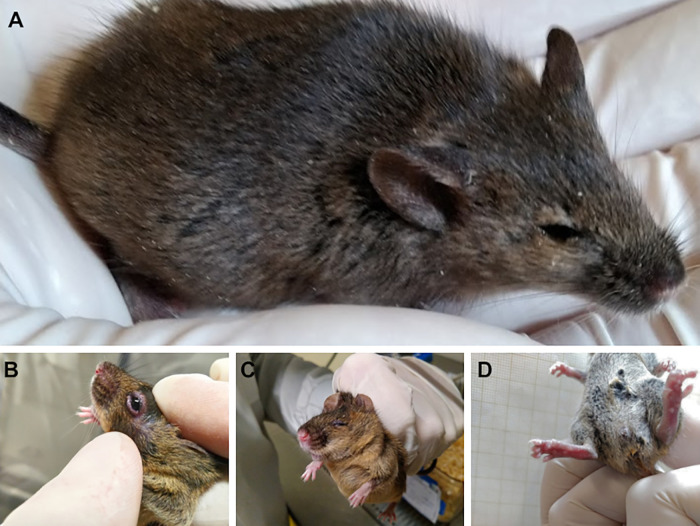
MAYV-infected A129 KO mice manifest overt disease phenotypes. **(A)** Animals expressed clinical signs of pain, including piloerection, backward-rotated ears, and orbital tightening. A129 KO also exhibited eye irritation (B) with blepharospasm (C) and pasty diarrhea **(D)**.

In general, infected mice exhibited clinical signs indicative of pain, including hunched posture, lethargy, piloerection, backward-rotated ears, orbital tightening, and bulging of the cheeks. Additionally, they exhibited eye irritation with tearing, secretion, blepharospasm, and pasty diarrhea (Fig 3). In this study, only two immunocompetent males exhibited mild and transient clinical signs, such as piloerection and lethargy, while no infection-related signs were observed in the control groups.

### Footpad swelling is evident in experimental MAYV infection

**Fig 4:**
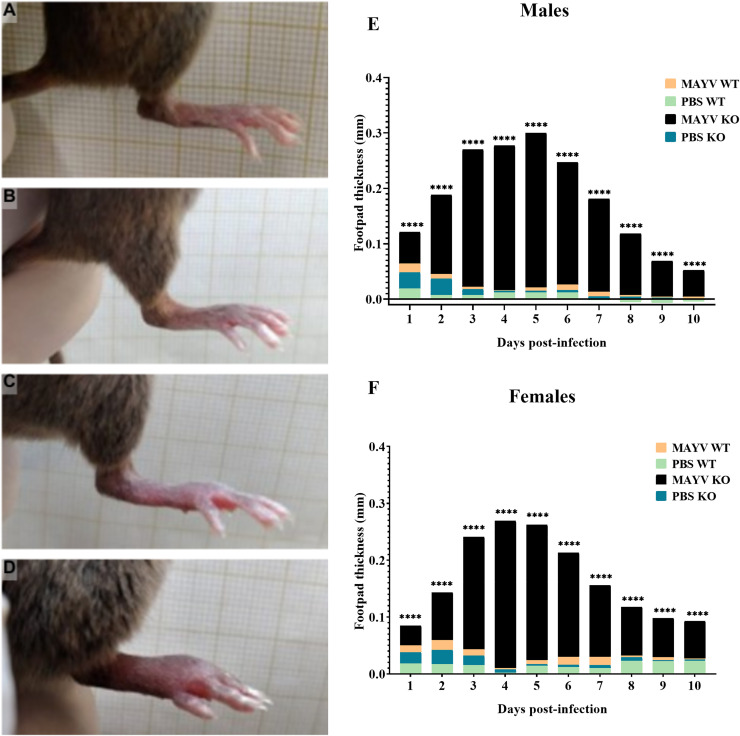
Footpad swelling is evident in MAYV-infected mice. Control wild type (A) and knockout (B) mice have no alterations in the footpad. Although WT mice have shown some evidence of edema **(C)**, the footpad swelling in the KO group (D) is more conspicuous. **(E)** The footpad thickness was significantly higher in infected KO males (MAYV KO) when compared to WT animals (MAYV WT) and controls (PBS KO) from 1 to 10 days post-infection (d.p.i.). **(F)** In females, the footpad thickness was also significantly higher in infected KO males (MAYV KO) when compared to WT animals (MAYV WT) and controls (PBS KO) during the entire evaluation period. Two-way ANOVA with Tukey’s multiple comparisons tests.

Footpad swelling was observed in MAYV-infected mice, particularly in immunodeficient animals (Fig 4A–4D). In KO males (Fig 4E) and females (Fig 4F), footpad thickness began to increase at 1 d.p.i., with the highest t values observed during the acute phase of infection. Significant differences between the infected groups (WT and KO) and within the KO group (infected and control) were observed throughout the entire evaluation period for both sexes. Despite the clinical evidence of edema in infected WT mice (Fig 4C, 4E, 4F), no significant differences in footpad thickness were found when compared to controls. Additionally, no alterations in the footpad thickness were observed in control animals during the evaluation period.

### Marked inflammatory response in the tibiotarsal joint is observed during MAYV infection

**Fig 5:**
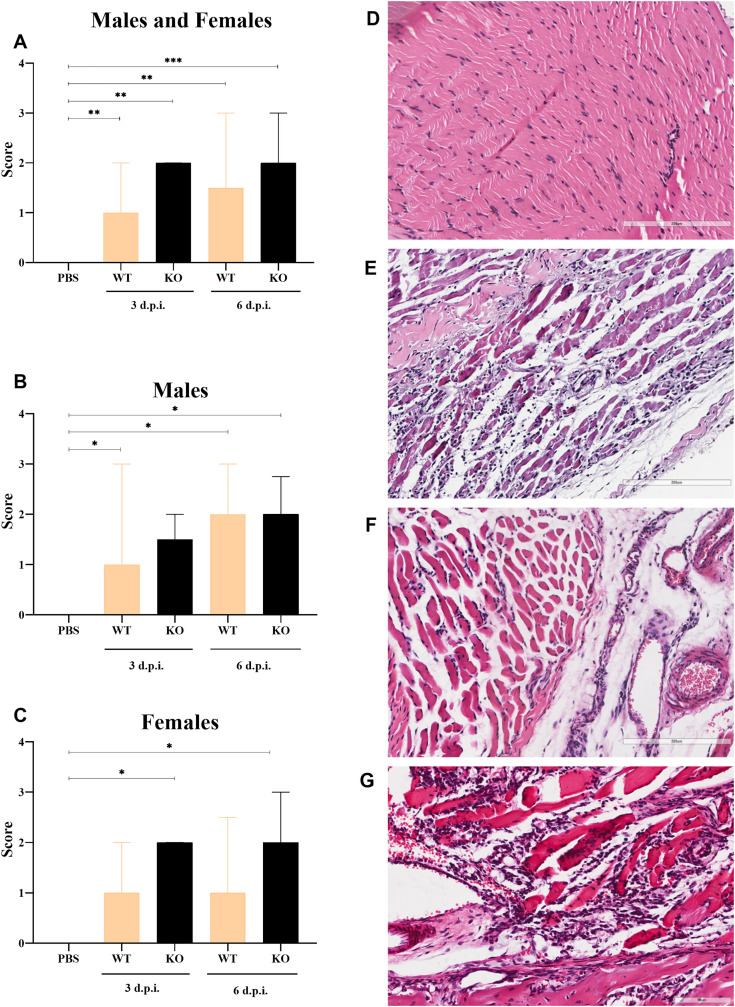
A marked inflammatory response is observed in the tibiotarsal joint of MAYV-infected wild-type and A129 KO mice. **(A)** Sex-independent evaluation of inflammatory cell infiltration scores in histopathological sections of the tibiotarsal joints and associated muscles show a significant increase in the inflammatory response in both wild-type (WT) and knockout (KO) mice when compared to controls (PBS) at 3 and 6 days post-infection (d.p.i.). **(B)** In male animals, inflammation scores were higher for WT mice compared to controls at 3 and 6 d.p.i., while for KO animals, significant differences were found only at 6 d.p.i. **(C)** In females, the inflammation scores were significantly higher than controls in the KO group. Kruskal-Wallis with Dunn’s multiple comparisons test. **(D)** Hematoxylin and eosin-stained slides show no alterations of the tibiotarsal joint muscles in control animals. **(E)** Lymphocytic inflammatory infiltrates are observed within the muscle fibers of WT males at 3 d.p.i. **(F)** Vasculitis was also observed in infected wild-type females at 3 d.p.i. **(G)** Degeneration of the muscle fibers associated with an intense mononuclear inflammatory response was observed in wild-type males at 6 d.p.i.

Histopathological evaluation of the tibiotarsal joints and associated muscles demonstrated higher inflammatory cell infiltration scores in MAYV-infected WT and KO mice compared to controls, regardless of sex and evaluation period (Fig 5A). Wild-type males showed significant differences at 3 and 6 d.p.i. compared to controls, while in the KO group, cell infiltration scores were significantly higher at 6. d.p.i. (Fig 5B). In contrast, significant differences in the inflammatory response were observed only in the KO MAYV-infected females (Fig 5C). The inflammatory response was characterized by lymphocytic infiltration in the muscle tissue (Fig 5D), synovial membrane, tendons, and ligaments (Fig 5E). Vasculitis (Fig 5F) and muscle fibers degeneration (Fig 5G) were also frequently observed in infected animals, regardless of sex and immunological profile.

### A129 KO females have hypernociception and loss of muscle strength

**Fig 6:**
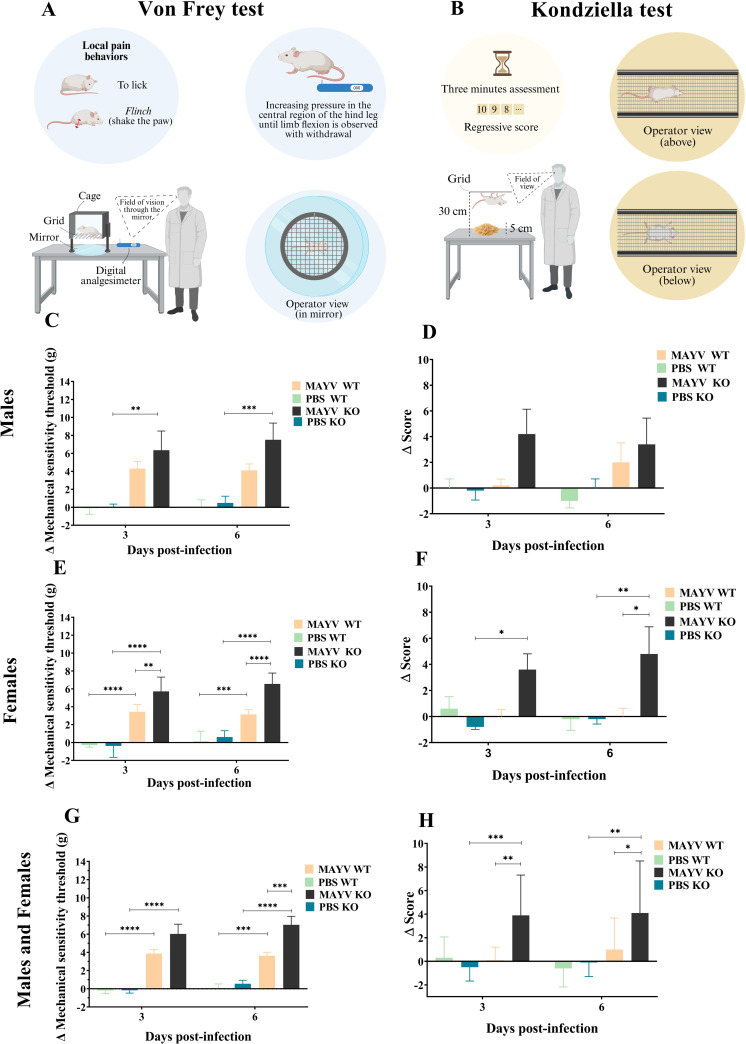
MAYV infection is associated with hypernociception and loss of muscle strength in A129 KO mice. **(A)** Schematic representation of the Von Frey test **(B)** Schematic representation of the Kondziella test. **(C)** Mechanical sensitivity thresholds are significantly lower in infected A129 KO (MAYV KO) males when compared to controls (PBS KO) at 3 and 6 days post-infection (d.p.i.). No differences were observed between infected wild-type (MAYV WT) and KO animals. **(D)** Infected females of both strains have higher hypernociception when compared to controls, and differences between infected WT and KO animals are significant at 3 and 6 d.p.i. **(E)** The sex-independent evaluation shows lower mechanical thresholds in KO compared to WT only at 6 d.p.i. **(F)** The Kondziella’s inverted screen test showed no significant alterations in the hind limb muscle strength of male mice. **(G)** MAYV KO females, in turn, have significant loss of muscle strength when compared to controls at 3 and 6 d.p.i. and to the WT group at 6 d.p.i. **(H)** The sex-independent evaluation demonstrates higher loss of muscle strength in MAYV KO when compared to MAYV WT and controls at both evaluation periods. Two-way ANOVA with Tukey’s multiple comparisons tests. This figure was made with Biorender.

The electronic Von Frey test revealed that female mice have greater sensitivity to mechanical stimuli in the hind limbs compared to males, regardless of genetic status (Fig 6). A significant reduction in the mechanical sensitivity threshold was observed in both immunodeficient and immunocompetent females at 3 and 6 d.p.i., with the lowest threshold values observed at 6 d.p.i. IFN-I knockout (KO) mice displayed hyperalgesia compared to both controls and immunocompetent animals. Wild-type females had reduced sensitivity thresholds at 3 and 6 d.p.i. compared to control, although to a lesser extent than that observed in KO animals. Mechanical hyperalgesia was also observed in KO males compared to controls at 3 and 6 d.p.i. (Fig 6B). Additionally, a sex-independent evaluation showed that differences in hypernociception between WT and KO mice were significant only at 6 d.p.i. (Fig 6C).

**Fig 7:**
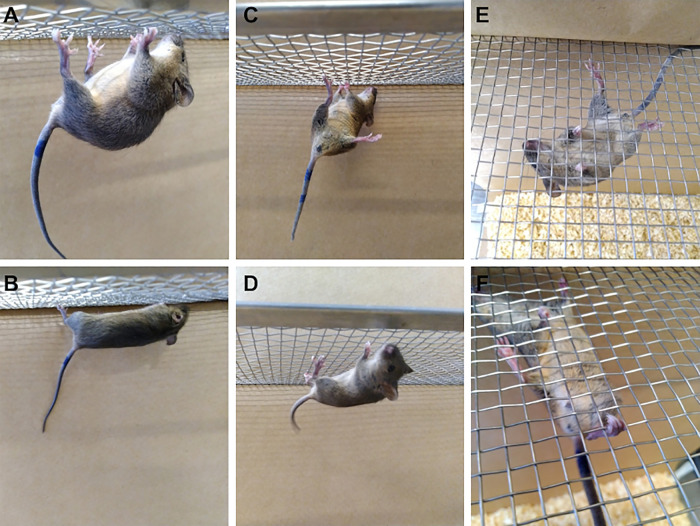
MAYV-infected A129 KO females had difficulty remaining attached to the grated platform during the Kondziella’s inverted screen test. **(A)** We observed an arching of the spine in infected female knockout mice during the Kondziella’s test when compared to controls **(B)**. **(C)** These animals also frequently changed limbs while grabbing the railing, a behavior known as sparing, which was not observed in control animals **(D)**. **(E)** MAYV-infected mice remained attached only by their fingertips and for short periods while controls grabbed the railing with their toes **(F)**.

During Kondziella’s inverted screen test, female KO mice exhibited more difficulty remaining attached to the mesh platform compared to males. These animals were unable to keep their hind limbs firmly attached to the railing, resulting in noticeable spine arching. MAYV-infected animals remained attached only by their fingertips and for short periods. They also frequently changed limbs while grabbing the railing, a behavior known as sparing (Fig 7). Furthermore, infected KO females showed greater loss of muscle strength compared to the WT group at 6 d.p.i. (Fig 6E). In the sex-independent evaluation, immunodeficient mice showed loss of muscle strength in both evaluation periods compared to controls, especially at 3 d.p.i. (Fig 6F).

### MAYV causes alterations in the platelet cell count of A129 KO mice

Red blood cell and white blood cell parameters from experimental animals are described in S3-S5 Tables. No significant differences were observed in the hematological profile of infected animals in relation to the evaluation period (3 and 6 d.p.i.) or when compared to controls, except for male A129 KO mice, which exhibited a significant increase in the platelet cell count (thrombocytosis) at 6 d.p.i. compared to A129 KO mice at 3 d.p.i. and to the control group at 6 d.p.i. (S2 Table).

### MAYV can be detected in several organs of infected mice

**Fig 8:**
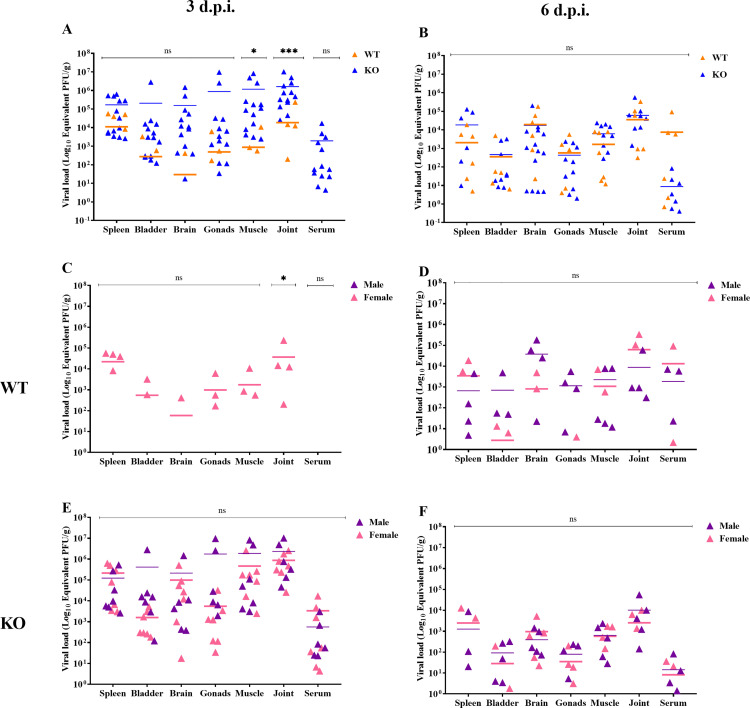
MAYV can be detected in several organs of infected mice. MAYV was detected in several organs and sera of wild-type (WT) and knockout (KO) animals at (A) 3 days post-infection (d.p.i.) and (B) 6 d.p.i. At 3 d.p.i., higher viral loads were observed in the tibiotarsal joint and associated muscles of KO when compared to WT mice. No significant differences between the strains were observed at 6 d.p.i. **(C)** At 3 d.p.i., viral loads were observed only in infected WT females, with no significant differences in viral loads between the sexes at 6 d.p.i. **(D)**. Also, no significant differences were observed between KO males and females at 3 (E) and 6 d.p.i. **(F)**. Two-way ANOVA with Tukey’s multiple comparisons tests and Bonferroni adjustment.

MAYV was detected in several organs and sera of both immunocompetent and immunodeficient animals at 3 and 6 d.p.i (Fig 8). At 3 d.p.i. (Fig 8A), higher viral loads were observed in KO compared to WT mice, particularly in the tibiotarsal joint and associated muscles. At 6 d.p.i. (Fig 8B), viral loads increased in the sera and spleen of WT and KO mice, respectively, although no significant differences were observed between the strains in this period. Additionally, we observed that WT females were more susceptible to MAYV infection at 3 d.p.i. (Fig 8C), exhibiting higher viral loads in the tibiotarsal joints compared to males (*p* = 0.032). No significant differences were observed between the sexes in the KO group (Fig 8E, 8F). Interestingly, viral load was detected in the brain and gonads of both WT and KO mice during evaluation periods.

## Discussion

The emergence of MAYV infections, associated with the vectorial competence of *Aedes aegypti* and the potential urbanization of the disease, has drawn global public health attention to the rapid development of vaccines and antiviral therapies. Appropriate animal models of the disease are necessary to evaluate the efficacy and safety of these prophylactic and therapeutic agents. Furthermore, a better understanding of the MAYV infection pathogenesis using a well-characterized animal model can provide complementary information on viral pathogenesis and transmission dynamics.

In this study, we conducted a thorough evaluation of MAYV infection in both immunocompetent (A129 WT) and type I IFN receptor-deficient animals (A129 KO). We demonstrated that the infection could not cause weight loss in animals infected with 10^4^ TCID_50_ of the virus, regardless of sex and immunological profile. Similar findings have been reported in another study using type 1 IFN receptor knockout mice (A129ABR), which showed no weight loss during the 6-day evaluation period after being infected with 10^5^ PFU of a human isolate from Peru [16]. On the other hand, 10^5^ PFU of the MAYV TR 4675 strain induced transient weight loss in 8-week-old IFN⍺R1^-/-^ mice and permanent weight loss in 11-day-old wild-type C57Bl/6 [17]. The IFN⍺R1^-/-^ mice were inoculated with 20 μL in the footpad and showed weight loss from 3 to 5 d.p.i. in the young C57Bl/6 mice, it occurred from 1 to 6 d.p.i. In the same study, however, 8-week-old RAG-1^-/-^ did not lose weight due to MAYV infection, nor did 21-day and 8-week-old C57BL/6 mice [16].

By evaluating the clinical score, we identified that the manifestation of phenotypes resulting from systemic infection is influenced by sex and immune status. Immunodeficient males had more evident clinical signs of the disease, in which the individual assessment demonstrated a high number of animals with moderate to severe phenotypes. Piloerection, lethargy, and hunched posture were observed in all animals that died from the infection. These clinical signs are commonly seen when animals are in pain, whether due to an infectious condition or other causes. These parameters are easily observed in infected mice [18–21].

MAYV infection in humans causes conjunctivitis, eye pain, and photophobia [22]. Similarly, A129 KO had severe ocular irritation with tearing, blepharospasm and purulent secretion. The same was observed in 15-day-old BALB/c mice, where ophthalmic signs were mostly unilateral but sometimes affected both eyes [20]. Considering that BALB/c mice have an intact immune system, we hypothesized that the infection succeeded in this model due to the young age of the study animals, which were infected before complete weaning. The strain of the MAYV (virus isolate TR4675, a genotype D strain) may have also contributed to the observed outcomes. In this sense, the virulence of circulating MAYV strains and the relationship with the genotypic profile need to be further explored. Additionally, BALB/c mice have a Th2-class skewed immune response, which is more efficient in controlling extracellular than intracellular viral pathogens [23]. With respect to eye irritation, this phenotype is frequently observed in *in vivo* models infected with other arboviruses, such as Zika virus (ZIKV) [23], CHIKV [24] and dengue virus (DENV) [25].

Footpad swelling has also been reproduced in murine models for arthritogenic alphaviral infections, especially CHIKV [26,27]. Considering that patients infected with MAYV develop similar symptoms to those infected with CHIKV [3,28,29], we evaluated edema formation in the hind limbs of MAYV-infected mice. We observed that male and female KO mice have more evident and persistent footpad edema than WT mice, with more severe swelling observed during the acute phase of the disease. Similar findings were obtained with 6–8-week-old C57BL/6 mice inoculated in the footpad with 10 μL of the TR4675 strain of MAYV [30,31] and with 8-week-old IFNAR^-/-^ mice inoculated with 20 μL of BeAr20290 strain (genotype L) [17].

In addition to analyzing the footpad edema, we have also conducted the histopathological analysis of the tibiotarsal joints of MAYV-infected animals and verified the presence of a lymphocytic inflammatory response in the synovial membrane, as well as edema, vasculitis, and degeneration of muscle fibers. Similar findings were reported elsewhere [17]. A study using inflammasome-deficient mice demonstrated that the pathogenesis of the MAYV-induced inflammatory response is associated with the activation of the NLRP3 inflammasome, production of reactive oxygen species, and potassium efflux, causing tissue edema and pain [30].

Furthermore, our results demonstrate that MAYV infection manifests more systemically in male mice than in females, in which local pain and loss of muscle strength are evident. The assessment of hypernociception and muscle strength showed that infected females have lower mechanical sensitivity thresholds and lower strength capacity in the hind limbs compared to males, especially at 6 d.p.i. Another study using the Von Frey test, 6–8-week-old C57BL/6 mice infected with 10^6^ of TR4675 strain manifested pain from 1 to 8 d.p.i. [30]. Regarding the Kondziella’s inverted screen test, this was the first study to consider the method for evaluating MAYV-related muscle strength loss, showing that it is a very sensitive assay to be used in animal models for arthritogenic arboviruses. Both the Von Frey and the Kondziella’s inverted screen allowed detection of functional differences in hypernociception and muscle strength that were identified by footpad thickness measurements in infected WT mice and controls. Furthermore, it must be considered that functional tests are more sensitive to detect injury than measuring paw diameter.

Despite the high-grade fever that is the hallmark of human disease, infected mice had hypothermia. Although this fact might seem paradoxical, we must consider that there is a systemic balance between fever and hypothermia. In this sense, when the cost of collateral damage is high, the organism temporarily tolerates the pathogen with the help of hypothermia while creating conditions to fight it again with the help of fever [32]. Considering that many animals that died in our study had severe illness, hypothermia may have acted as a defense mechanism for the organism itself.

Regarding the hematological alterations associated with alphaviral diseases, leukopenia is commonly observed in the acute phase of human infection [11,33,34]. In another *in vivo* study, the inoculation of 2 × 10^3^ PFU of a WT MAYV strain into interferon type I receptor-deficient A129 mice increased global and differential leukocyte counts [16]. This was not observed in our study with the MT/SINOP/210/2011 virus strain. Moreover, infected male KO mice showed a significant increase in the platelet count at 6 d.p.i. when, in fact, thrombocytopenia is common in arbovirus infections [35,36]. This increase in platelet count may be an overproduction from the body’s recovery process. In this sense, further studies with other circulating MAYV strains are warranted to characterize the hematological profile of infected animals.

The viral load examination demonstrated the systemic dissemination of MAYV in all infected animals, regardless of sex and immunological profile. As expected, we obtained high viral titers in the tibiotarsal joints and associated muscles. MAYV is an arthritogenic virus, and other studies with similar pathogens, such as the alphavirus CHIKV, have demonstrated high viral concentrations in the joints and muscles of experimental models [26,37]. These results are in accordance with the clinical findings in MAYV-infected patients, who, like CHIKV-infected subjects, suffer from severe pain and inflammation of the joints and muscles [3,28,29].

High viral titers were also detected in the gonads, spleen, and brain. Importantly, we verified no reduction in the viral load in these organs at 6 d.p.i. in immunocompetent mice. The possibility of new variants adapted to these systems raises the possibility that the MT/SINOP/210/2011 strain could be neurovirulent and sexually transmitted, which makes this arbovirus a serious healthcare concern. Our findings are similar to ZIKV studies showing its tropism for neural cells and the genitourinary system; as a consequence, ZIKV has been known to cause neuropathogenic conditions and to be sexually transmitted [37–40].

In this study, we demonstrated that A129 KO mice are a suitable model for *in vivo* investigations on MAYV. Our tests indicate that animals infected with the MT/SINOP/210/2011 strain manifested the disease’s main phenotypes similar to the Mayaro fever observed in humans. However, it is important to consider the limitations of the selected animal model, especially regarding the targeted species’ strains. A129 KO mice, for example, are susceptible to viral infections and can be widely used in viral pathogenesis assays. Nevertheless, their limitation in terms of immune response should be carefully evaluated for vaccine testing, as it might affect the interpretation of efficacy results. Alternatively, this strain could be used as a lethal model since infected animals succumbed to the disease.

On the other hand, clinical signs of the disease are less evident in wild-type 129S1 mice when evaluated for a short period after infection. Considering that these animals are as immunocompetent as most human patients, further research on the characterization of this model is therefore warranted. Alternatives to date consist of using young animals with vulnerable immune systems and administering immunosuppressants in adult animals. Serial passage of the virus in immunocompetent mice to obtain an adapted strain should also be considered.

Finally, this work is the first to characterize an *in vivo* model infected with the Brazilian strain of MAYV (MT/SINOP/210/2011). We demonstrated significant sex-conditioned differences in disease manifestation, in which males had more systemic signs of infection while females had more local pain. We have also standardized the Von Frey and Kondziela’s inverted screen test to assess nociception and loss of muscle strength, respectively, in MAYV-infected mice, thus warranting further application of this method in other animal models of viral arthritis. The established model will be instrumental in the testing and development of new countermeasures against this emerging arbovirus.

## Materials and methods

### Ethics statement

This *in vivo* study was approved by the Ethics Commission on Animal Use from the Federal University of Uberlândia on September 18^th^, 2020 (CEUA-UFU, #029/2020). All protocols were in accordance with the provisions of the Brazillian Law n. 11.794, of October 8^th^, 2008, the Decree n. 6.899 of July 15^th^, 2009, and the resolutions of the National Council for Control of Animal Experimentation (CONCEA).

### Cell and virus

Vero CCL-81 cells provided by the Aggeu Magalhães Institute (Fiocruz Pernambuco) cell bank were used in the cultivation and titration of the MAYV strain (MT/SINOP/210/2011, GenBank: KF305672.1) isolated from a patient in Sinop, Mato Grosso, Brazil [41]. Vero cells were cultured with culture medium DMEM (Dulbecco’s Modified Eagle’s Medium) supplemented with 10% fetal bovine serum and 1% penicillin/streptomycin (10,000 U/ml) at 37°C and 5% CO_2_. For the viral stock preparation, cells were infected with about 80–90% confluency and maintained with DMEM supplemented with 2% fetal bovine serum and 1% penicillin/streptomycin (10,000 U/mL) at 37°C and 5% CO2 for 48h. The culture supernatant was stored at -80°C, and the TCID_50_/mL method defined the viral titer.

### Mouse model

Experimental units consisted of A129 KO (B6.129S2-*If*nar1**^tm1Agt^/Mmjax, MMRRC stock #32045) and A129 WT mice (129S1/SvImJ) mice provided by the Rodents Animal Facilities Complex from the Federal University of Uberlândia (REBIR-UFU), Brazil. The A129 KO mice are interferon I receptor-deficient and, therefore, susceptible to viral infections [41], while WT animals are immunocompetent. The animals were specific pathogen-free (SPF) males and females, 21 days old and weighing 15 to 20g. They were kept in 32 x 20 x 21 cm micro-isolator cages on ventilated racks, with autoclaved chips of *Pinus ellioti* as bedding material. Irradiated food and autoclaved water were provided *ad libitum.* Animals were maintained on a light/dark cycle of 12/12 hours under controlled temperature and humidity conditions (22°C ± 2°C/ 55% ± 5%).

### Pathogenicity assessment

A129 WT and A129 KO mice, both males and females, were anesthetized with 4% isoflurane via inhalation and then subcutaneously inoculated with either 10^5^ TCID50 of MAYV (infected group) in 10 μL of phosphate-buffered saline (PBS) or PBS alone (control group) into the hind footpad on the right side of the animals.

Animals were monitored for survival (n = 52), weight loss (n = 40), and clinical signs of disease (n = 40) after infection (S6 Table). A numerical score was assigned to a group of clinical signs, as described: (0) healthy, (1) mild signs of lethargy, piloerection, (2) increased lethargy, piloerection, eye irritation, diarrhea, hunched posture, (3) increased lethargy, piloerection, eye irritation, diarrhea, hunched posture (4) increased lethargy, piloerection, eye irritation, diarrhea, hunched posture, limited mobility, (5) minimal mobility and inability to eat or drink water. In addition, the severity of the phenotypes was individually evaluated using a numerical visual scale, as follows: (1) mild, (2) medium, and (3) severe.

### Body temperature measurement

The body temperature of the experimental animals (n = 40) was measured before infection and at 3 and 6 d.p.i. using an infrared digital thermometer (Hetaida, China). The measurement point was the abdomen, with three measurements per animal being performed, considering the mean for data tabulation. The measurements were conducted in the morning in an environment free of artificial refrigeration.

### Hematological profiling

Blood samples from three animals per group were collected at 3 and 6 d.p.i. After intraperitoneal administration of ketamine (100 mg/kg) and xylazine (10 mg/kg), approximately 200μL were collected into ethylenediaminetetraacetic acid (EDTA) tubes from the retro-orbital plexus. Following this, animals were euthanized via cervical dislocation and necropsy. Samples were processed in auto-hematology analyzer Hematoclin 2.8 VET (Quibasa-Bioclin, Belo Horizonte, Brazil) to obtain the red blood cell count (RBCC), hemoglobin (Hgb), hematocrit (Hct), mean corpuscular volume (MCV), mean corpuscular hemoglobin (MCH), mean corpuscular hemoglobin concentration (MCHC), white blood cell count (WBCC), platelet cell count (PCC) and the red blood cell distribution width (RDW). In addiction, manual differential leukocyte counts were performed on blood smears stained with panoptic fast stain kit (LaborClin, Santa Bárbara, Brazil)

### Histopathological analysis

After necropsy, the right tibiotarsal joints of five animals per group were fixed with 10% neutral buffered formalin and decalcified with 10% EDTA, pH 7. Tissues were then subjected to histological processing, and 3µm sections were stained with hematoxylin and eosin (H&E). Microscopy slides were scanned using Aperio AT2 (Leica Biosystems, Nussloch, Germany) at 400x magnification. The quantification of the inflammatory cell infiltration was performed at 3 and 6 d.p.i. by two independent evaluators based on the following score: (0) without infiltrates; (1) light infiltrates; (2) moderate infiltrates; (3) severe infiltrates, as previously reported [42]. A final score was assigned to each group based on the evaluation.

### Tissue processing and viral RNA extraction

Samples from the spleen, bladder, brain, gonads, tibiotarsal joints, associated muscles, and sera obtained during blood sample collection and necropsy were stored at −80°C until processing. PBS buffer was added to the samples in a 9:1 ratio. The samples were agitated at 30 Hz for 5 minutes in the TissueLyser (Qiagen, USA) to promote cell and tissue disruption, releasing the viral RNA. Then, the beads were removed using a magnetic retriever, and the tubes were centrifuged at 14.000 rpm for 10 minutes to obtain the supernatant, which was used for viral RNA extraction. Viral RNA was extracted from 140 μl of mouse samples supernatant using the QIAamp Viral RNA Mini Kit (Qiagen, Germany) following the manufacturer protocol. After extraction, the RNAs were stored at −80°C until the RT-qPCR was performed.

### Viral load quantification

The presence of MAYV was confirmed using the RT-qPCR with primers and protocols previously described [43], with minor modifications. Each reaction was prepared using the QuantiNova Probe RT-PCR Kit (Qiagen, USA) following the manufacturer’s protocol in a total volume of 10 μl. Negative and positive controls (RNA extracted from the supernatant of cells infected with MAYV) were applied to validate the results. For sample quantification, a 10-fold standard curve was built with a detection limit of 10 PFU/mL. Cycling steps were performed as follows: (1) 45°C for 15 minutes, (2) 95°C for 5 minutes, (3) 45 cycles of 95°C for 3 seconds and (4) 55°C for 30 seconds. All experiments were conducted using the Applied Biosystems QuantStudio 5 real-time PCR systems (Applied Biosystems, USA). Data analysis was performed using QuantStudio v1 software.

### Evaluation of the mechanical nociceptive threshold

Mechanical sensitivity in the hind limbs was assessed using the Von Frey electronic test [44]. Briefly, mice were placed in 12 × 20 × 17 cm acrylic cages on a wire grid floor. After the twenty-minute acclimatization period, a trained evaluator applied increasing pressure in the central region of the hind paw until the observation of limb flexion with withdrawal. Tests were performed by the same operator at 0, 3, and 6 d.p.i., always in the afternoon. Three measurements were taken from each experimental unit. Test results were quantified as the variation (Δ) in the nociceptive threshold (g), subtracting the averaged three baseline values expressed by the averaged three values obtained after infection.

### Muscle strength assessment

Experimental animals were submitted to Kondziella’s inverted screen test [45] to assess the loss of muscle strength at 0, 3, and 6 d.p.i., always in the morning. A grid-shaped platform was positioned 30 cm above the bench, which was provided with a 5 cm bedding of *Pinus ellioti* chips. Animals were then hung upside down to the grid for 3 minutes. An initial score of ten points was assigned to each animal, with a 1-point decrease in the score for each fall during the evaluation period.

### Statistical analyses

All analyses were conducted in GraphPad Prism 8.2.1 (GraphPad, San Diego, California, USA), with α = 5%. Differences in the body temperature, weight, clinical score, footpad swelling, inflammatory cell infiltration, and mechanical nociceptive threshold between the infected and control groups were verified with two-way ANOVA and Tukey’s *post hoc* test. Differences in the hematological parameters between infected and control groups were verified with Kruskal-Wallis and Dunn’s *post hoc* test. Viral quantifications were compared in relation to genetic background and sex at different times of infection, using a two-way ANOVA test with Bonferroni adjustment. Survival analyses were performed using the Mantel-Cox log-rank test.

## Supporting information

S1 Table Number of infected animals per group showing clinical scores from 0 to 5 throughout the 10-day evaluation period. (DOC)

S2 Table Analysis of red blood cell parameters in male A129 WT and KO mice infected with MAYV. (DOCX)

S3 Table Analysis of white blood cell and platelet cell parameters in male A129 WT and KO mice infected with MAYV. (DOCX)

S4 Table Analysis of red blood cell parameters in female A129 WT and KO mice infected with MAYV. (DOCX)

S5 Table Analysis of white blood cell and platelet cell parameters in female A129 WT and KO mice infected with MAYV. (DOCX)

S6 Table Distribution of the experimental groups in the pathogenicity assay. (DOCX)
