# Supplementary material for: A comprehensive murine clinical model for development of countermeasures and studying Mayaro virus infection
Source: PLoS Negl Trop Dis. 2025 Jul 31;19(7):e0013333. doi: 10.1371/journal.pntd.0013333 (PMC12349698; doi:10.1371/journal.pntd.0013333)
Supplement: S1 Table — (DOC) [file pntd.0013333.s001.DOC]

**S1 Table.** Number of infected animals per group showing clinical scores from 0 to 5 throughout the 10-day evaluation period.

|  | **Experimental groups, n** | | | | | | | | | | | | | | | | | | | | | | | | | | |
| --- | --- | --- | --- | --- | --- | --- | --- | --- | --- | --- | --- | --- | --- | --- | --- | --- | --- | --- | --- | --- | --- | --- | --- | --- | --- | --- | --- |
| **Time**  **(d.p.i.)** | **Males** | | | | | | | | | | | | |  | **Females** | | | | | | | | | | | | |
|  | **MAYV WT** | | | | | |  | **MAYV KO** | | | | | |  | **MAYV WT** | | | | | |  | **MAYV KO** | | | | | |
|  | **Clinical score** | | | | | |  | **Clinical score** | | | | | |  | **Clinical score** | | | | | |  | **Clinical score** | | | | | |
|  | **0** | **1** | **2** | **3** | **4** | **5** |  | **0** | **1** | **2** | **3** | **4** | **5** |  | **0** | **1** | **2** | **3** | **4** | **5** |  | **0** | **1** | **2** | **3** | **4** | **5** |
| **1** | 10 | 0 | 0 | 0 | 0 | 0 |  | 7 | 3 | 0 | 0 | 0 | 0 |  | 10 | 0 | 0 | 0 | 0 | 0 |  | 4 | 6 | 0 | 0 | 0 | 0 |
| **2** | 10 | 0 | 0 | 0 | 0 | 0 |  | 3 | 6 | 1 | 0 | 0 | 0 |  | 10 | 0 | 0 | 0 | 0 | 0 |  | 3 | 6 | 0 | 1 | 0 | 0 |
| **3** | 10 | 0 | 0 | 0 | 0 | 0 |  | 2 | 3 | 3 | 2 | 0 | 0 |  | 10 | 0 | 0 | 0 | 0 | 0 |  | 3 | 4 | 2 | 1 | 0 | 0 |
| **4** | 6 | 4 | 0 | 0 | 0 | 0 |  | 1 | 1 | 1 | 7 | 0 | 0 |  | 10 | 0 | 0 | 0 | 0 | 0 |  | 3 | 4 | 2 | 1 | 0 | 0 |
| **5** | 10 | 0 | 0 | 0 | 0 | 0 |  | 1 | 1 | 2 | 6 | 0 | 0 |  | 10 | 0 | 0 | 0 | 0 | 0 |  | 4 | 4 | 2 | 0 | 0 | 0 |
| **6** | 10 | 0 | 0 | 0 | 0 | 0 |  | 4 | 2 | 2 | 2 | 0 | 0 |  | 10 | 0 | 0 | 0 | 0 | 0 |  | 4 | 5 | 1 | 0 | 0 | 0 |
| **7** | 10 | 0 | 0 | 0 | 0 | 0 |  | 2 | 4 | 3 | 1 | 0 | 0 |  | 10 | 0 | 0 | 0 | 0 | 0 |  | 4 | 6 | 0 | 0 | 0 | 0 |
| **8** | 10 | 0 | 0 | 0 | 0 | 0 |  | 4 | 4 | 2 | 0 | 0 | 0 |  | 10 | 0 | 0 | 0 | 0 | 0 |  | 6 | 3 | 1 | 0 | 0 | 0 |
| **9** | 10 | 0 | 0 | 0 | 0 | 0 |  | 5 | 4 | 1 | 0 | 0 | 0 |  | 10 | 0 | 0 | 0 | 0 | 0 |  | 6 | 3 | 1 | 0 | 0 | 0 |
| **10** | 10 | 0 | 0 | 0 | 0 | 0 |  | 6 | 2 | 2 | 0 | 0 | 0 |  | 10 | 0 | 0 | 0 | 0 | 0 |  | 6 | 4 | 0 | 0 | 0 | 0 |

n, number of animals; PBS WT, control wild-type mice; PBS KO, control knockout mice; MAYV WT: infected wild-type mice; MAYV KO: infected knockout mice; d.p.i., days post-infection.
