# Supplementary material for: A comprehensive murine clinical model for development of countermeasures and studying Mayaro virus infection
Source: PLoS Negl Trop Dis. 2025 Jul 31;19(7):e0013333. doi: 10.1371/journal.pntd.0013333 (PMC12349698; doi:10.1371/journal.pntd.0013333)
Supplement: S2 Table — (DOCX) [file pntd.0013333.s002.docx]

**S2 Table.** Analysis of red blood cell parameters in male A129 WT and KO mice infected with MAYV.

| **Parameters** | **Experimental groups, median (min – max)** | | | | | | | | | | | |
| --- | --- | --- | --- | --- | --- | --- | --- | --- | --- | --- | --- | --- |
|  | **PBS WT** | |  | **PBS KO** | |  | **MAYV WT** | |  | **MAYV KO** | | *p*-value* |
|  | **3 d.p.i** | **6 d.p.i** |  | **3 d.p.i** | **6 d.p.i** |  | **3 d.p.i** | **6 d.p.i** |  | **3 d.p.i** | **6 d.p.i** |  |
| **RBCC (10^6^/uL)** | 11.5 (1.6) | 11.8 (3.9) |  | 9.8 (2.6) | 10.7 (5.4) |  | 10.0 (1.0) | 9.5 (1.5) |  | 10.2 (6.1) | 9.3 (4.2) | ns |
| **Hemoglobin (g/dL)** | 19.9 (1.7) | 17.7 (8.3) |  | 16.3 (5.0) | 18.8 (7.3) |  | 17.1 (2.5) | 16.2 (2.2) |  | 17.3 (7.6) | 16.2 (5.8) | ns |
| **Hematocrit (%)** | 59.3 (8.7) | 54.2 (15.5) |  | 50.6 (15.1) | 56.2 (22.2) |  | 51.8 (4.7) | 49.7 (6.1) |  | 52.3 (30.2) | 48.0 (18.7) | ns |
| **MCV (fL)** | 51.0 (2.7) | 50.9 (5.5) |  | 51.7 (3.2) | 52.0 (8.1) |  | 51.3 (5.3) | 55.6 (6.5) |  | 51.5 (2.4) | 51.1 (13.5) | ns |
| **MCH (pg)** | 16.9 (1.5) | 17.6 (3.3) |  | 16.8 (1.3) | 17.1 (2.5) |  | 16.4 (2.4) | 18.7 (3.1) |  | 17.1 (13.3) | 17.2 (35.8) | ns |
| **MCHC (g/dL)** | 32.2 (2.3) | 33.1 (6.6) |  | 32.3 (0.5) | 32.8 (1.4) |  | 32.3 (1.8) | 33.4 (2.6) |  | 32.9 (8.9) | 33.1 (15.9) | ns |
| **RDW (%)** | 12.7 (1.5) | 12.7 (4.2) |  | 12.8 (3.4) | 13.3 (5.7) |  | 12.5 (2.7) | 15.1 (4.0) |  | 13.9 (2.2) | 12.8 (21.8) | ns |

PBS WT, control wild-type mice; PBS KO, control knockout mice; MAYV WT: infected wild-type mice; MAYV KO: infected knockout mice; d.p.i., days post-infection; RBCC, red blood cell count; MCV, mean corpuscular volume; MCH, mean corpuscular hemoglobin; MCHC, mean corpuscular hemoglobin concentration; RDW, red cell distribution width; ns, not significant; *Kruskal-Wallis with Dunn’s post-hoc test, *p* <.0.05.
