# Supplementary material for: A comprehensive murine clinical model for development of countermeasures and studying Mayaro virus infection
Source: PLoS Negl Trop Dis. 2025 Jul 31;19(7):e0013333. doi: 10.1371/journal.pntd.0013333 (PMC12349698; doi:10.1371/journal.pntd.0013333)
Supplement: S3 Table — (DOCX) [file pntd.0013333.s003.docx]

**S3 Table.** Analysis of white blood cell and platelet cell parameters in male A29 WT and KO mice infected with MAYV.

| **Parameters** | **Experimental groups, median (min – max)** | | | | | | | | | | | |
| --- | --- | --- | --- | --- | --- | --- | --- | --- | --- | --- | --- | --- |
|  | **PBS WT** | |  | **PBS KO** | |  | **MAYV WT** | |  | **MAYV KO** | | *p*-value* |
|  | **3 d.p.i** | **6 d.p.i** |  | **3 d.p.i** | **6 d.p.i** |  | **3 d.p.i** | **6 d.p.i** |  | **3 d.p.i** | **6 d.p.i** |  |
| **Leucocyte x (10^3^/uL)** | 5.4 (4.0) | 7.6 (8.4) |  | 8.3 (6.0) | 6.3 (21.4) |  | 4.9 (5,5) | 5.8 (2.4) |  | 2.5 (4.2) | 4.4 (5.4) | ns |
| **Lymphocyte (%)** | 77.0 (6.0) | 76.5 (20.0) |  | 74.0 (16.0) | 79.0 (6.0) |  | 80.0 (30.0) | 64.0 (44.0) |  | 67.0 (53.0) | 66.0 (42.0) | ns |
| **Segmented neutrophils (%)** | 19.0 (5.0) | 19.5 (21.0) |  | 20.0 (18.0) | 16.0 (8.0) |  | 20.0 (27.0) | 29.0 (16.0) |  | 30.0 (56.0) | 29.0 (46.0) | ns |
| **Band neutrophils (%)** | 0.0 (0.0) | 0.0 (0.0) |  | 0.0 (0.0) | 0.0 (0.0) |  | 0.0 (4.0) | 0.0 (4.0) |  | 0.0 (6.0) | 0.0 (2.0) | ns |
| **Eosinophils (%)** | 1.0 (2.0) | 1.0 (2.0) |  | 1.0 (2.0) | 1.0 (1.0) |  | 0.0 (1.0) | 2.0 (4.0) |  | 1.0 (9.0) | 1.0 (2.0) | ns |
| **Basophils (%)** | 0.0 (0.0) | 0.0 (1.0) |  | 0.0 (3.0) | 0.0 (2.0) |  | 0.0 (0.0) | 0.0 (2.0) |  | 0.0 (2.0) | 1.0 (2.0) | ns |
| **Platelets x (10^3^/uL)** | 605.0 (480.0) | 713.0 (130.0) |  | 728.0 (450) | 647.0^a^ (242.0) |  | 668.0 (375.0) | 767.0 (240.0) |  | 531.0^b^ (710.0) | 1078.0^ab^ (545.0) | .01 |

PBS WT, control wild-type mice; PBS KO, control knockout mice; MAYV WT: infected wild-type mice; MAYV KO: infected knockout mice; d.p.i., days post-infection; ns, not significant; *Kruskal-Wallis with Dunn’s post-hoc test, *p* <0.05. Similar lowercase letters (a or b) denote the absence of significant statistical differences; different lowercase letters denote the presence of significant statistical differences.
