# Supplementary material for: A comprehensive murine clinical model for development of countermeasures and studying Mayaro virus infection
Source: PLoS Negl Trop Dis. 2025 Jul 31;19(7):e0013333. doi: 10.1371/journal.pntd.0013333 (PMC12349698; doi:10.1371/journal.pntd.0013333)
Supplement: S4 Table — (DOCX) [file pntd.0013333.s004.docx]

**S4 Table.** Analysis of red blood cell parameters in female A129 WT and KO mice infected with MAYV.

| **Parameters** | **Experimental groups, median (range)** | | | | | | | | | | | |
| --- | --- | --- | --- | --- | --- | --- | --- | --- | --- | --- | --- | --- |
|  | **PBS WT** | |  | **PBS KO** | |  | **MAYV WT** | |  | **MAYV KO** | | *p*-value* |
|  | **3 d.p.i** | **6 d.p.i** |  | **3 d.p.i** | **6 d.p.i** |  | **3 d.p.i** | **6 d.p.i** |  | **3 d.p.i** | **6 d.p.i** |  |
| **RBCC (10^6^/uL)** | 10.6 (1.1) | 9.2 (3.4) |  | 10.1 (1.41) | 9.3 (6.4) |  | 10.6 (5.4) | 10.1 (5.1) |  | 9.7 (1.6) | 10.1 (1.7) | ns |
| **Hemoglobin (g/dL)** | 17.5 (2.1) | 16.5 (31.1) |  | 17.1 (2.1) | 16.9 (10.4) |  | 17.6 (6.8) | 16.3 (5.9) |  | 16.4 (2.7) | 17.0 (13.0) | ns |
| **Hematocrit (%)** | 53.9 (5.0) | 47.0 (16.3) |  | 51.9 (6.8) | 51.4 (24.6) |  | 53.8 (17.9) | 51.0 (16.7) |  | 46.8 (8.0) | 50.7 (8.4) | ns |
| **MCV (fL)** | 51.0 (3.6) | 51.8 (3.9) |  | 51.4 (2.1) | 55.5 (12.5) |  | 50.7 (15.1) | 50.3 (14.2) |  | 50.5 (0.2) | 50.4 (3.7) | ns |
| **MCH (pg)** | 16.5 (1.7) | 17.7 (2.2) |  | 17.0 (0.6) | 18.8 (2.2) |  | 16.5 (3.4) | 16.0 (3.8) |  | 16.7 (0.5) | 17.0 (0.7) | ns |
| **MCHC (g/dL)** | 32.4 (1.6) | 33.8 (3.6) |  | 33.1 (0.4) | 32.8 (5.9) |  | 32.5 (2.5) | 31.9 (1.5) |  | 33.2 (0.9) | 33.9 (1.7) | ns |
| **RDW (%)** | 12.7 (1.0) | 13.8 (4.6) |  | 12.4 (1.7) | 13.5 (3.2) |  | 12.7 (2.5) | 12.7 (2.1) |  | 13.0 (1.2) | 12.5 (2.4) | ns |

PBS WT, control wild-type mice; PBS KO, control knockout mice; MAYV WT: infected wild-type mice; MAYV KO: infected knockout mice; d.p.i., days post-infection; RBCC, red blood cell count; MCV, mean corpuscular volume; MCH, mean corpuscular hemoglobin; MCHC, mean corpuscular hemoglobin concentration; RDW, red cell distribution width; ns, not significant; *Kruskal-Wallis with Dunn’s post-hoc test, *p* <0.05.
