# Supplementary material for: A comprehensive murine clinical model for development of countermeasures and studying Mayaro virus infection
Source: PLoS Negl Trop Dis. 2025 Jul 31;19(7):e0013333. doi: 10.1371/journal.pntd.0013333 (PMC12349698; doi:10.1371/journal.pntd.0013333)
Supplement: S5 Table — (DOCX) [file pntd.0013333.s005.docx]

**S5 Table.** Analysis of white blood cell and platelet cell parameters in female A129 WT and KO mice infected with MAYV.

| **Parameters** | **Experimental groups, median (range)** | | | | | | | | | | | |
| --- | --- | --- | --- | --- | --- | --- | --- | --- | --- | --- | --- | --- |
|  | **PBS WT** | |  | **PBS KO** | |  | **MAYV WT** | |  | **MAYV KO** | | ***p*-value*** |
|  | **3 d.p.i** | **6 d.p.i** |  | **3 d.p.i** | **6 d.p.i** |  | **3 d.p.i** | **6 d.p.i** |  | **3 d.p.i** | **6 d.p.i** |  |
| **Leucocyte x (10^3^/uL)** | 5.6 (4.2) | 3.9 (8.6) |  | 4.9 (8.6) | 2.7 (7.7) |  | 4.1 (3.2) | 3.5 (6.3) |  | 2.3 (5.5) | 4.6 (2.0) | ns |
| **Lymphocyte (%)** | 80.0 (16.0) | 76.0 (19.0) |  | 82.0 (22.0) | 66.0 (14.0) |  | 70.0 (38.0) | 72.0 (16.0) |  | 66.0 (44.0) | 72.0 (19.0) | ns |
| **Segmented neutrophils (%)** | 22.0 (11.0) | 22.0 (18.0) |  | 16.0 (17.0) | 29.0 (9.0) |  | 24.0 (38.0) | 22.0 (18.0) |  | 23.0 (52.0) | 22.0 (24.0) | ns |
| **Band neutrophils (%)** | 0.0 (0.0) | 0.0 (0.0) |  | 0.0 (1.0) | 0.0 (0.0) |  | 0.0 (4.0) | 0.0 (6.0) |  | 0.0 (6.0) | 0.0 (4.0) | ns |
| **Eosinophils (%)** | 1.0 (1.0) | 0.0 (2.0) |  | 1.0 (1.0) | 1.0 (4.0) |  | 1.0 (6.0) | 1.0 (4.0) |  | 0.0 (2.0) | 3.0 (4.0) | ns |
| **Basophils (%)** | 0.0 (1.0) | 0.0 (0.0) |  | 0.0 (2.0) | 0.0 (1.0) |  | 2.0 (2.0) | 0.0 (0.0) |  | 0.0 (3.0) | 2.0 (4.0) | ns |
| **Platelets x (10^3^/uL)** | 805.0 (245.0) | 847.0 (394.0) |  | 730.0 (170.0) | 340.0 (466) |  | 738.0 (526.0) | 626.0 (392) |  | 606.0 (114.0) | 748.0 (214.0) | ns |

PBS WT, control wild-type mice; PBS KO, control knockout mice; MAYV WT: infected wild-type mice; MAYV KO: infected knockout mice; d.p.i., days post-infection; ns, not significant; *Kruskal-Wallis with Dunn’s *post-hoc* test, *p* <0.05.
