# Supplementary material for: A comprehensive murine clinical model for development of countermeasures and studying Mayaro virus infection
Source: PLoS Negl Trop Dis. 2025 Jul 31;19(7):e0013333. doi: 10.1371/journal.pntd.0013333 (PMC12349698; doi:10.1371/journal.pntd.0013333)
Supplement: S6 Table — (DOCX) [file pntd.0013333.s006.docx]

**S6 Table.** Distribution of the experimental groups in the pathogenicity assay.

| **Treatment** | **Survival** | |  | **Weight loss** | |  | **Clinical signs** | |  |
| --- | --- | --- | --- | --- | --- | --- | --- | --- | --- |
|  | **WT** | **KO** |  | **WT** | **KO** |  | **WT** | **KO** |  |
|  | **♂/♀** | **♂/♀** |  | **♂/♀** | **♂/♀** |  | **♂/♀** | **♂/♀** |  |
| **MAYV** | 26 | 26 |  | 20 | 20 |  | 20 | 20 |  |
| **PBS** | 26 | 26 |  | 20 | 20 |  | 20 | 20 |  |

WT, wild-type mice; KO: knockout mice; ♂, male mice; ♀, female mice; MAYV, infected group; PBS, control group.
